# Parental Intervention Program for Preschool children with Rare Diseases – a mixed methods evaluation of parents’ experiences and utility

**DOI:** 10.1186/s13023-023-02935-8

**Published:** 2023-10-17

**Authors:** Gry Velvin, Vigdis Johnsen, Ingeborg Beate Lidal, Ellen Berg

**Affiliations:** 1 TRS National Resource Centre for Rare Disorders, Sunnaas Rehabilitation Hospital, Nesodden, Norway; 2 The Institute of Public Health, Norwegian Knowledge Centre for the Health Services, Oslo, Norway; 3 School of Sport Sciences, Department of Teacher Education and Outdoor Life Studies, Oslo, Norway

**Keywords:** Mixed-methods evaluation, Parental intervention, Preschool children, Rare diseases, Transition from kindergarten to school

## Abstract

**Background:**

The purpose of this study was twofold: (i) To assess the parents’ experiences and perception of participating in a “Parental Intervention Program for Preschool children with Rare Diseases” (PIPP-RDs). (ii) To evaluate which elements of the PIPP-RDs that the parents emphasized as important for improving their health literacy related to facilitating the transition of their children from kindergarten to school.

**Method:**

A mixed methods evaluation study was conducted ten and eleven months post-intervention, integrating an online quantitative survey combined with individual semi-structured interviews. Twenty-two parents participated in individual interviews, of these 18 also responded to the online questionnaire survey.

**Results:**

All parents that participated in this study reported that the information conveyed at the program was of great value and utility, 88% reported significantly alleviated stress associated to their child`s school-start, 84% indicated had improved the school-home collaboration and 84% reported that it had encouraged them to establish contact with the school prior to school commencement. From the qualitative data five main themes emerged: (i) Competence and Knowledge Acquisition, (ii) Becoming more Prepared and Relaxed, (iii) Achieved Realistic Expectations, (iv) Enhanced Communication Skills, (v) Increased Health Literacy and Self-Efficacy. The evaluative findings suggest that this invention program has notably improved the parents’ aptitude for school interaction, enhanced the adaptions according to children`s needs for accommodations, and has provided reassurance in the school-home collaboration. Parents also described increased self-confidence and self-efficacy in managing the school start for children with RDs.

**Conclusion:**

The highly positive response of participating in PIPP-RDs may not only reflect the merits of the program`s content, but also underscore the significant needs for such support during the transition to school for parents of children with RDs. Comparable initiatives, oriented towards enhancing the health literacy and empowering the parents, are anticipated to yield similarly favourable results. We argue that intervention program amalgamate pertinent information, group discourse, and workshops on school-related issues, alongside opportunities for parents to meet other parents in similar situations.

**Supplementary Information:**

The online version contains supplementary material available at 10.1186/s13023-023-02935-8.

## Introduction

Despite the fact that many children with rare diseases (RDs) experience challenges in the educational sphere, and that parents play a decisive role in the transition from kindergarten to school, few studies have investigated the needs of parents of children with RDs. and their potential to increase knowledge and health literacy to manage this transition process [1].

### Rare diseases

RDs are medical conditions that affect 1in 2,000 individuals in Europe and are defined in the US as condition affecting fewer than 200,000 people (*1:1600) at any given time [2–4]. It is estimated that there are approximately 7,000 distinct RDs, affecting between 18 and 30 million Europeans and 300 million individuals worldwide [3, 4], many of whom are children [5]. Approximately 72% of RDs have a genetic origin, and 70% have childhood onset (4). Notably, approximately 95% of RDs currently have no approved treatment [2, 4] create significant challenges for affected individuals and society as a whole. The impacts of RDs are often unexplored and range from psychological and physical symptoms, seriously compromising participation in education, work and daily life [1, 2, 4, 5]. The United Nations (UN) acknowledges that individuals living with RDs and their families may be psychological, socially, and economically vulnerable throughout their life course, facing specific challenges in several areas, including schooling and education [5]. The UN also express concerns that people living with RDs and their families may be at greater risk of being disproportionately affected by stigma, discrimination, and social exclusion, and that one major barrier is the lack of knowledge and awareness of RDs [5].

Caring for children with RDs may be demanding and stressful [6, 7], potentially intensified by the rarity of the condition. Children with RDs often have chronic and complex medical conditions that necessitate multidisciplinary follow-up [7, 8]. The burden of parents has been reported to have negative impact on their physical and psychological health and may cause practical and financial difficulties [7–9].

### The rationale for the study in the context of art of state

Despite the great heterogeneity of different RDs, parents seem to face many similar problems related to the rarity of the disease [6–11], such as lack of information and competence, limited clinical experiences and published research to inform school and interdisciplinary professionals, and lack of evidence-based practice. The lack of knowledge and information about the disease, its prognosis, and the children’s needs and development may pose challenges for both parents and school personnel in facilitating appropriate adjustments as the children embark on their schooling journey [1, 12–14]. Teachers may feel inadequately prepared for the task of both educating students with RDs and ensuring that the teaching environment adequately meet the children’s needs [1, 12, 14].

Few studies have addressed the challenges and opportunities for children with RDs at school, as well as the importance of the parental role in educational settings [1]. Despite limited research, studies indicate that children with RDs may have more complex educational needs than those of their peers with more common disabilities [1, 15–17]. Families may experience stigma and isolation within educational settings, and professionals often perceive themselves as inadequately skilled when educating children with RDs [1, 14].

Despite the widely accepted understanding that parental involvement positively impacts students’ achievement [18–22], no studies have been identified dealing with school- related intervention program for empowering and preparing parents of preschool children with RDs to facilitate the transition from kindergarten to school.

### The parental intervention program for Preschool children with Rare Diseases

In Norway, both the National Health Services and the school system are public entities, with education being compulsory for all children aged between 6 and 16 years. Typically, education legislation encourages the inclusion of children with disabilities and chronic diseases into mainstream schools, as stipulated by the Norwegian Education Act [23]. While most children with RDs attend mainstream public schools, many parents concurrently face challenges, particularly during the transition phase from kindergarten to school.

TRS [24] is a National Resource Centre for Rare Diseases (TRS) in Norway that serves people with congenital skeletal and connective tissue disorders, spina bifida and limb deficiency. The users of the centre are people affected by these disorders, their families, health personnel and other professionals working with these patient groups. The core activities of TRS encompass individual counselling, development and implementation of various intervention programs, collaboration with healthcare, educational, and social services, as well as research, and knowledge dissemination.

In the clinical setting, TRS frequently encountered parents expressing a need for additional support during the transition process from kindergarten to school for their children with RDs. Based on the clinical experiences, parents expressed needs, pertinent research, and a theoretical framework of situated learning [25], the TRS developed the Parental Intervention Program for Preschool children with Rare Diseases (PIPP-RDs) in 2002. Since then, this intervention program has been offered to parents of preschool children with RDs annually. The overall goal of the PIPP-RDs is to enhance parents` health literacy and empower them to facilitate the transition from kindergarten to school, thereby contributing to the enhancement of school experiences and educational opportunities for children with RDs.

The program emphasizes preparing parents for school commencement by informing them about opportunities and potential challenges, strategies for effective communication, rights and obligations, accommodations and assistive devices, and how to navigate and address challenges within the school system to ensure their children’s needs in the context of the school system. The PIPP-RDs is structured as a four-day workshop, specifically designed for parents of preschool children with various RDs. The intervention program is scheduled in January, seven months prior to the children’s entrance into primary school.

Each year, approximately 10 to 18 families participate in the program. The families reside at a course campus, where social gatherings are organized in the evenings. During the day, the parents engage in an intensive program comprising brief presentations by different professionals on various topics related to the education system, guided group discussions, practical advice sessions, and workshops. The content of the PIPP-RDs emphasizes both provision of information and facilitating discussion on necessary adaptations to address physical, practical, and learning challenges at school. Group discussions encompass a range of topics, from pedagogical and psychological considerations to the social and emotional aspects associated with having a preschool child with RDs related to the transition from kindergarten to school.

A flipped classroom approach is utilized [26], with participants receiving online lectures two weeks prior to the PIPP-RDs. This strategy encourages the parents to reflect on their informational needs, their anticipated challenges associated with starting school, and factors pertinent to home-school collaboration. Flipped classroom strategies are grounded in blended learning, stimulating parents to actively participate in the program and directly connect its content with their everyday lives. Consequently, parents become more actively involved and adequately prepared prior to their engagement in the PIPP-RDs. However, the extent of preparation is at the discretion and based on voluntariness of the parents.

During the PIPP-RDs four-day intensive program, parents are first introduced to the program’s overall purpose and practical content. Then, the parents engage in group discussions on various topics. Group-based interventions are important parts of the program, because this method has been found to increase parental self-efficacy [27]. The topics in the PIPP-RDs are described in Table 1.

**Table 1:** The issues emphasized in the PIPP-RDs

**The issues emphasized in the PIPP-RDs**:
- Expectations and opportunities for starting school - Educational adaptation - Adapted and inclusive Physical Education - Strategies for informing and communication with others about their child’s disease - Collaboration between school and home - Embracing differences - Navigating challenges of having a child with a RD - Practical adaptations for the children’s needs - Perspectives on enhancing the children’s transition to school Throughout the program, parents work on developing a schedule plan for how they will facilitate the transition process for their child, from kindergarten to school.

Based on an understanding that parents of children with rare diseases often face a challenging life situation, careful consideration was given to enable optimal adherence. Thereby, emphasizing the program`s accessibility by ensuring it was not overly demanding or extensive.

## The objective of the study

The purpose of our article was twofold: (i) To assess parents’ experiences and perception of participating in a PIPP-RDs 10 and 11 months post-intervention, and (ii) to discern elements of the intervention that seem to enhance parents’ health literacy, associated with the transition of their child from kindergarten to school. We also wanted to gain in-depth knowledge about their experiences and their views of the impact of participating:

We posted the following questions:

1. What are the parents’ experiences of participating in the PIPP-RDs, and do they perceive any benefits related to their role of facilitating their children’s transition from kindergarten to school?
2. What specific advantages or disadvantages do the parents emphasize regarding participation in PIPP-RDs, and which aspects of PIPP-RDs do they highlight as most important?
3. How do the parents perceive the significance of their participation in the PIPP-RDs for school-home collaboration?

## Materials and methods

### Design and recruitment

This research employed a mixed-methods approach, encompassing both a quantitative online questionnaire survey and semi-structured individual interviews. A total of thirty-six families, from every health region in Norway, participated in the PIPP-RDs during 2017 and 2018. From each family, one parent was invited to participate in this study.

**Fig. 1:**
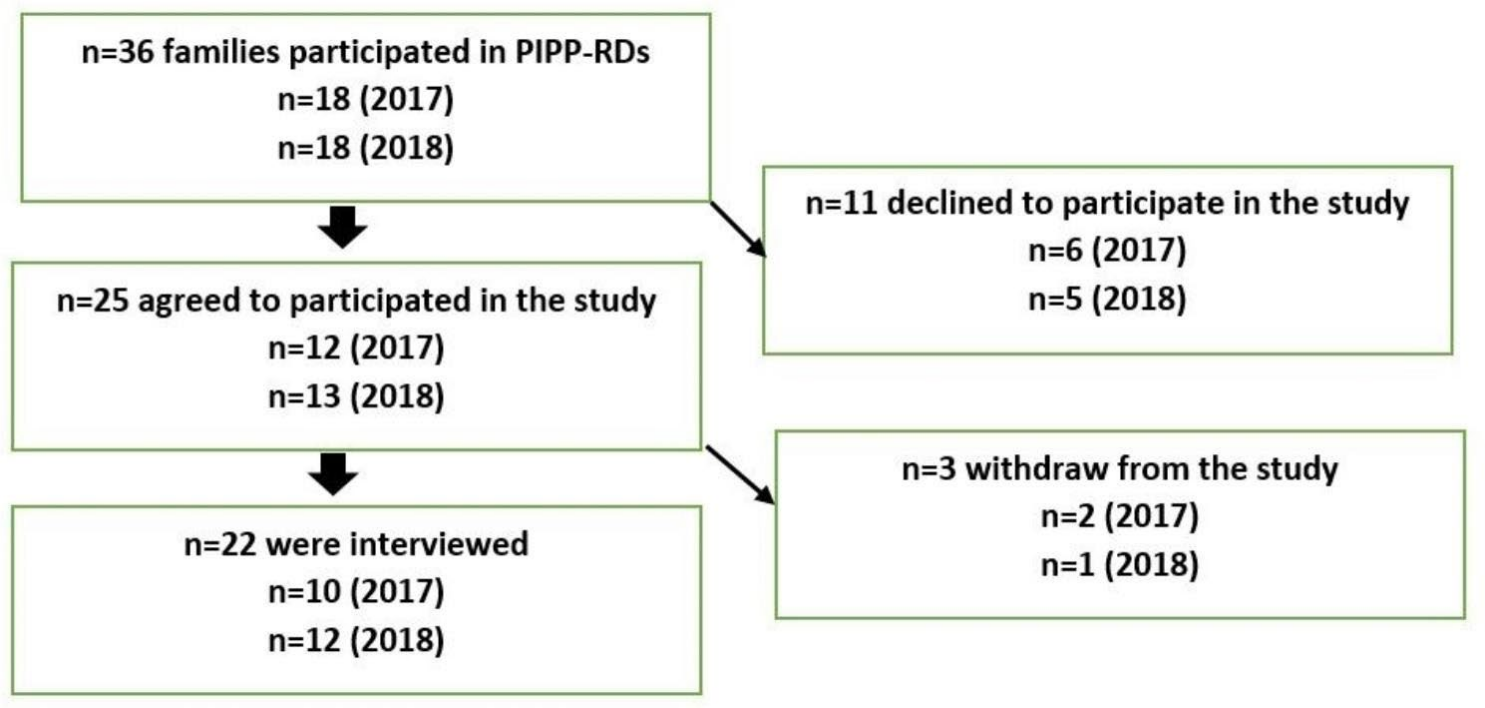
The inclusion process. The flow chart for the participant selection process

Among the 25 initial consents for individual interviews, three were retracted prior to the commencement of the study because they felt too overloaded to participate in the study. The inclusion process is depicted in the flow chart as outlined in Fig. 1.

A total of twenty-two parents participated in the qualitative part of the study and were interviewed in December ( 11 months post intervention and 4 months after starting school). Of these 18 also completed the online questionnaire survey in November (10  months post-intervention and 3 months after staring school) of the corresponding year. The time points for activities are described in Table 2. Findings from time point 4 and 5 (T4/T5) is outlined in this paper (Table 2).

**Table 2:** Time points for activities

Activities	Time-point
Flipped-classroom approach	T1 (8 January)
Intervention Programme (PIPP-RDs)	T2 (22–25. January)
School start-up for the children with RDs	T3 (August)
Questback quantitative questionnaire to the parents	T4 (November)
Semi-structured interviews with the parents	T5 (December)

### Quantitative online questionnaire part of the study

#### The questionnaire instrument

We considered adapting established tools for measuring health literacy or self-efficacy, but a problem is that very few validated instruments measure these issues concerning parents aiding their children’s` school transition, especially for children with disabilities and rare diseases. Therefore a study specific questionnaire was developed. This study-specific instrument comprised five demographic queries and 15 inquiries designed to measure the parents’ perceptions and experiences regarding the impact of the PIPP-RDs associated to the transition process from kindergarten to school. An additional eight questions focused on the parents` evaluation of the topics emphasized within the program. Measurement scales utilized in the questionnaire included a dichotomous scale (yes/no) and a 5-point Likert scale (ranging from 5 = very useful/satisfactory and 1 = very useless/unsatisfactory). The questionnaire also incorporated open-ended free-text options to capture more nuanced responses (see supplementary file 1). The electronic questionnaire was distributed to the parents using an online platform.

#### Analysis

Data collection was facilitated by the Questback online research platform, an online web program that ensures complete anonymization of respondents’ answers. The same platform was leveraged for data analysis [28]. In accordance with the study objectives, survey data are presented descriptively, utilizing proportions and figures in both the textual and graphical format. As the target population is restricted to some specific RDs, we anticipated a relatively small sample size, and consequently, we did not employ advanced statistical analysis. The results are presented in the form of the questions, and the questions have been translated from Norwegian to English in collaboration with a professional translator. We followed the Strengthening the Reporting of Observational Studies in Epidemiology (STROBE) guidelines [29].

### Qualitative in-depth semi-structured telephone-interview

#### The semi-structured interviews

The semi-structured telephone interviews focused on the parents’ perceptions of participating in the PIPP-RDs; their experiences of the program, particularly the utility in facilitating their child’s schooling (see supplementary file 2). The interviews were conducted by two researchers, and ranged from 25 to 45 min in duration. The interviews were audiotaped and transcribed verbatim.

#### Analysis

The transcriptions were thoroughly read by two researchers to identify emergent categories and themes. Thematic analysis, guided by the recommended four-step process by Braun & Clark [30], was utilized to identify patterns within the data material. Throughout the qualitative data analysis process, two researchers convened discussions, and an additional researcher was engaged as needed to resolve discrepancies in the interpretations. All four researchers collaboratively participated in step 3 and 4, and the writing up for the analysis [30]. We followed the recommended consolidated criteria for reporting qualitative research, COREQ [31]. The analysis process is illustrated in Table 3.

**Table 3:** Four-step thematic analysis

Steps in thematic analysis	Content of steps in the analysis
**Step 1. Overall impression of the material** • The authors (GV,VJ) thoroughly read the material independently of each other	• Formed an overview of the material • Started the process of identifying themes and used analysis for theme development
**Step 2. Coding-creating the first codes** • The authors (GV,VJ) identified pattern and codes in the material independent of each other	• Developed codes from preliminary themes • Coded and developed elements of meaning and the sorted and grouped these into meaning units.
**Step 3. Text condensation and identifying themes** • The authors (GV,VJ) condensed the material independently of each other • The authors (GV,VJ) assessed critical relevant themes together and subsequently involved the rest of the research group (IL,EB) in the discussion	• Searched to pattern in the material • Critically discussed themes, codes and categories • Reached a consensus on themes and categorisations
**Step 4. Synthesising and naming themes** • The authors (GV,VJ, IL, EB) discussed the theme names and codes in order to reach consensus	• Parts of the text were regrouped • Presented and analysed text with prominent content and meaning • Kept an analytic distance and discussed • Linked underlying meaning in the themes and categories to each other to create main themes

### Combining the data

Both the questionnaire and the semi-structured interview guide were developed in collaboration with designers of the program, the intervention program holders, and based on clinical experiences, relevant research. In addition we employed an integrated theoretical approach that integrate Epstein`s theory of Parental Involvement in Education [32], which delineates six distinct roles for parents in fostering a partnership between schools, families, and communities. This theory was harmoniously combined with the Strength perspective [33], which emphasizes recognizing and building upon the strength and assets of a child with a disability, rather than concentrating solely on their deficits or limitations. To gain a better understanding and a more solid foundation for developing the study specific tools for examining health literacy, we drew upon Nutbeam`s typology [34, 35], encompassing the three dimensions of health literacy: functional, interactive/communicative, and critical.

The aim was to ensure that the tools effectively captured the relevance of the intervention, reflecting its significance for the parents and grounded it in a solid theoretical foundation. This was conducted by a convergence between the quantitative and qualitative approaches.

The two sub-studies were first analysed individually. Following this a mixed methods approach [36, 37] was employed, with the primary emphasis placed on analysing, integrating and, synthesizing the results from the sub-studies. The objective of synthesizing the data from the sub-studies was to provide a more comprehensive and deeper understanding of the parents’ experiences and perspectives on participating in the PIPP-RDs. While the results from the survey and the semi structured interviews are presented separately in the findings, the discussion section integrates and triangulates the two data sources [37].

### Ethical considerations

This article is part of a larger study about Parental intervention programs for preschool children with RDs. The study was approved by the Data Protection Authority at Oslo University Hospital, Norway (17/20,647), ensuring that the study met appropriate ethical guidelines. Prior to participation, comprehensive written and oral information concerning the study was furnished to all potential participants, and written informed consent was duly obtained. Ethical aspects were meticulously addressed throughout all phases of the research process. Taking into account that the participants were the parents of children with RDs in Norway who could be easily identifiable in this context, a detailed description of the study group has not been presented. All information that may reveal the identity of the participants has been removed from the quotations. In the qualitative part of the study, each parent was assigned a code consisting of a number from 1 to 22 to ensure anonymity. This coding system was applied throughout all quoted materials.

## Results

### The respondents

The participants (n = 22) displayed a mean age of 38 years, spanning range of 25 to 55 years. The sample comprised 68% mothers and 32% fathers. In terms of family structure, 47% of the families had one child, 42% had two children, and 11% were parents to three children. Notable, no families had more than one child diagnosed with a rare disease. The children included in this study were diagnosed with a diverse range of conditions, including osteogenesis imperfecta, arthrogryposis multiplex congenital, spina bifida, multiple osteochondromas, short stature, congenital limb deficiency, Marfan syndrome, and Ehlers-Danlos syndrome.

### Online quantitative survey results

**Fig. 2:**
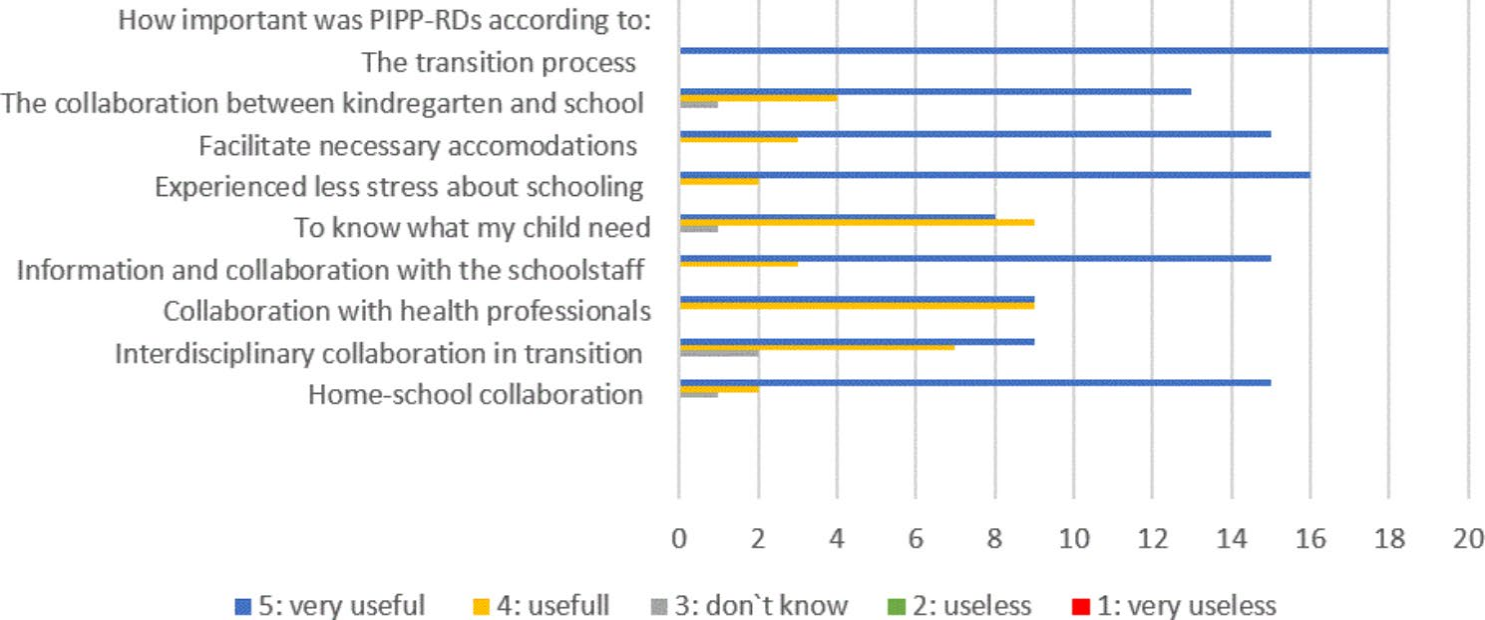
Parents’ utility of participating. Parents perceived utility of participating in PIPP-RDs

All parents that participated in this study (100%) reported that the information presented during the PIPP-RDs had been very useful for the transition process. Additionally, 88% found it very useful for alleviating stress related to their child’s school start, and 84% found it very useful for school-home collaboration. Only 44% found it very useful for increasing their knowledge about their child`s needs”, while 50% found it useful, and 5% were uncertain (don`t know). No parents reported the intervention program as useless or very useless, but some were uncertain (don`t know) about its usefulness in certain aspects, as detailed in Fig. 2.

All parents (100%) reported that PIPP-RDs met their needs for knowledge and information. Additionally, 88% found it easier to inform others about their children’s diseases after attending, and 84% initiated contact with their respective schools due to the information they received. Also, 88% reported that it facilitated informing others about their children’s needs. All parents would recommend PIPP-RDs to other parents of children with RDs and all emphasized the benefit of meeting other parents.

**Fig. 3:**
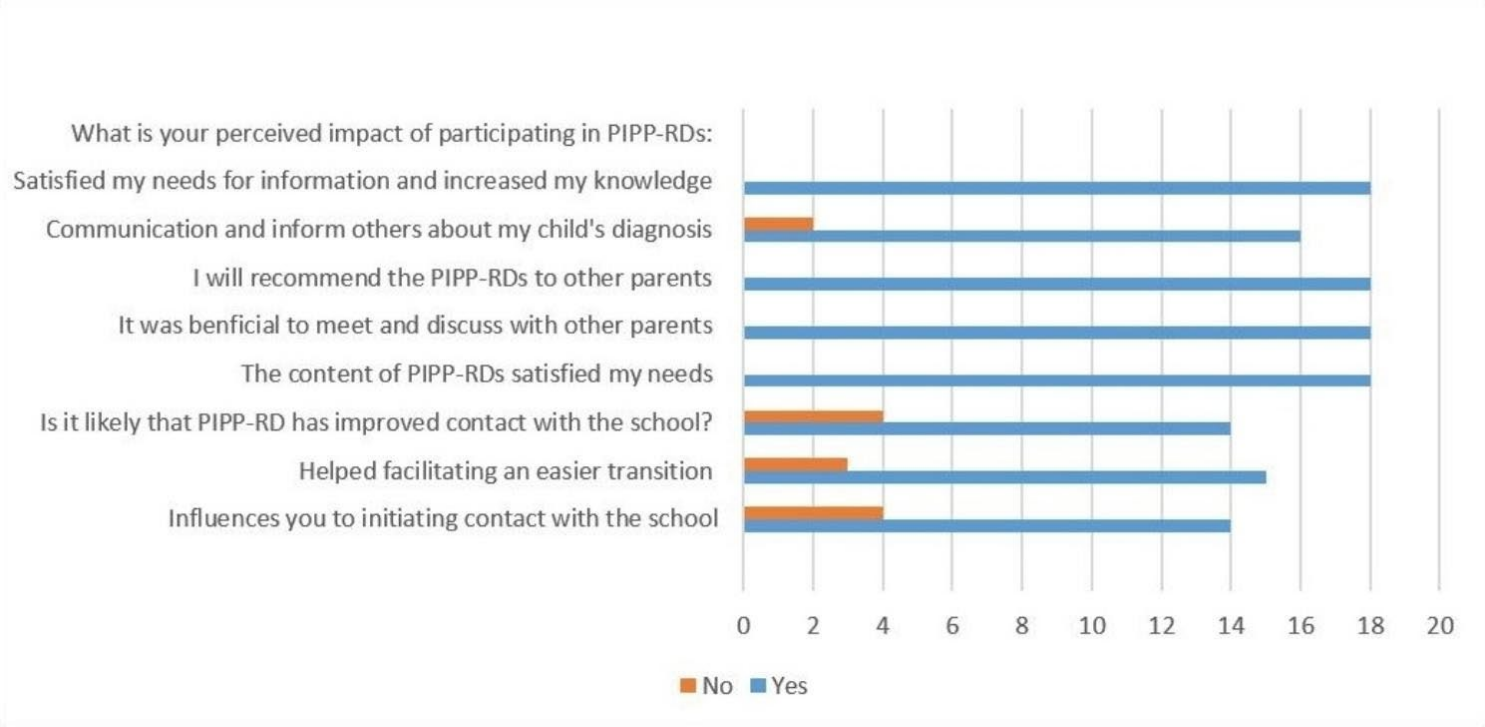
Parents perceived impact of participate

### The qualitative interview results

From the thematic analysis five interrelated main themes emerged, which illustrate the parents’ assessment of the program`s impact: (i) Competence and Knowledge Acquisition (ii) Becoming more Prepared and Relaxed (iii) Achieved Realistic Expectations (iv) Enhanced Communication Skills (v) Increased Health Literacy and Self-Efficacy. These themes describe various aspects of barriers, facilitators, and strategies that the program promoted for the parents’ facilitation of their children’s transition to school. While some themes clearly resonate across all parents’ experiences, other are notably more specific to the individual parents` unique situations.

#### Competence and knowledge acquisition

Numerous parents underscored their previously limited knowledge and familiarity with the school system, thereby attesting the invaluable utility of the information and topics disseminated via the PIPP-RDs.

One mother stated: “It was useful in many ways, and I think all information about all the issues at the program were of relevance…… and I felt I had much more knowledge afterwards” (Parent 1).

The parents valued practical advices, utilitarian aids, discussions about pragmatic solutions, and information on legal entitlement and the school system, all viewed as critical for smoother transition management. Certain parents indicated that the acquisition of current information and a more comprehensive understanding of the school system augmented their perceived competence. Some parents reported that gaining current information and a deeper understanding of the school system enhanced their perceived competence. Additionally, many highlighted that clear information on relevant school contacts encouraged them to initiate communication with the school immediately after completing the PIPP-RDs.

One parents, notable a father, underscored the pertinence of the program`s content: ”Indeed, it was valuable! Navigating this chaotic landscape is challenging, thus the program has proven significantly beneficial. Specifically, the practical advices and insight into effective strategies have been enlightening. Understanding our entitlements, our potential actions, and what we can do and how to do it” (Parent 3).

In summary, the detailed information provided about the school system seems to have enhanced parents’ perceived competence, empowering them to take a more proactive role in their child’s transition to school. A significant majority reported positive experiences when initiating early collaboration with the school staff. Many parents, who had little prior knowledge of the school system, found the program`s information and topics immensely useful. Concrete advice, practical aids, solution-focused discussion, and information about statutory rights and the school system were consistently highlighted as important components of the intervention program.

#### Being more prepared and relaxed

Many parents reported that before participating in the PIPP-RDs, they had either avoided contemplating how to facilitate their child’s schooling or believed this responsibility mainly fell on the school. Some voiced concerns about their child’s integration, potential for bullying, and uncertainty regarding how to inform the school and other students about their child’s rare condition.“

One mother remarked: “I worried a lot and was overwhelmed with concern for my child. The program, however, helped me in several ways, making me feel more comfortable and less apprehensive about my child beginning at school” (Parent 9).

Some parents underscored their lack of experiences in managing these challenges, with some expressing feeling of isolation. They reported having no previous encounters with other parents of children with RDs prior to the PIPP-RDs, underscoring the value of meeting with the other parents within the program.

A mother noted “The opportunity to share my thoughts and emotions with other parents was incredibly valuable… it fostered a sense of community. We no longer feel so alone and isolated” (Parents 7).

The group discussions and informal conversations with other parents in sharing feelings and thoughts, was also emphasized as important. The majority maintained contact with the other parents they met during the program, with some even establishing joint Facebook groups.

Several parents professed that their participation in the program and talking to other parents in similar situations had significantly reduced their stress levels, rendering them more relaxed and empowered.

One mother shared: “In a sense, we were able to breathe a sigh of relief, because we have been so apprehensive about school start… the program and meeting others bolstered my confidence. I am uncertain if we could have navigated this without the program” (Parent 5).

Another mother reflected similar sentiments: “I was plagued by numerous worries, but talking to others provided me with considerable aid. I feel that I`ve grown more comfortable and less worried about my child beginning at school” (Parent 9).

As part of the PIPP-RDs, parents were aided in developing a dynamic plan to facilitate their child`s transition into school, encompassing strategies for collaboration with the school system and health services. This planning phase honed their consciousness of their child`s needs, and guided them in advocating their child`s strengths and vulnerabilities effectively, determining the right communication partners and timings. Some integrated this plan into the individual care plan addressing their child`s specific needs, strengths, and areas of participation.

One father shared: “We`ve leveraged the information from the program in facilitating schooling … our plan was particularly crucial as it guided us on “how” and “when” of implementation” (Parent 2).

Many utilized the plan as a checklist when collaborating with teachers and health services for schooling arrangements. Notably, no parents reported any negative responses from school staff or health services when executing this plan.

A mother`s experience with the plan resonates in the following statement: “It`s been excellent. I feel significantly more prepared and alert to maintain things as they should be… the fusion of the schedule plan in the program was a smart move” (Parent 11).

#### Achieved realistic expectations

As mentioned above, many parents claimed that before the PIPP-RDs, they had unrealistic expectations and demands on the school due to their limited knowledge of how to facilitate the transition process. Some parents reported that gaining a more comprehensive understanding of their formal rights and the opportunities to discuss potential challenges related to the school transition process helped shape more realistic and reasonable expectations. They believed they had developed an increased understanding and tolerance for the teachers’ challenges, and some claimed they had experienced a cognitive shift towards a more realistic, collaborative, solution-oriented approach, rather than confrontational.

As one father described: “I believe I`ve evolved. I no longer feel the need to “win the battles”…I try to be more objective and have more understanding, then we will find good solutions together” (Parent 14).

#### Enhanced communication skills

Parents also claimed that their communication skills had changed after participating in the PIPP-RDs. Some described becoming more effective in articulating their child’s needs, and referred to information learned during the program to support their points. Others also noted an expansion of vocabulary and the ability to communicate in a less emotional and more professional way when talking about their children.

One father stated: “Before the program, everything felt chaotic… I was unsure about who I could contact. But now I know who to talk with and how I can communicate in a way they understand” (Parent 3).

Parents reported that school staff exhibited interest and attentiveness towards the information the parents had gleaned from the program. Some even observed an enhanced sense of respects.

One father said: “I think we cooperate very well… the school staff are so nice and they try to do as best as they can… but the system is not always so easy to fight” (Parent 19).

#### Increased health literacy and self-efficacy

Overall, the qualitative interviews indicated that many parents found their children’s transition to school to be highly satisfactory, with some describing it as “surprisingly well”. They underscored the potential impact of early school preparation and collaboration with the school staff, and acknowledged their own significant role in the process. Shifts from being worried to feeling of increased competence, preparedness, relaxation and realism were emphasized by several as pivotal outcomes of participation in the program. Some even noted enhanced ability to balance between making demands to the school and being humble and understanding.

A mother expressed: “I felt more competent and empowered, and I knew how to do it. That was good” (Parent 13).

Many parents seemed to exhibit an augmented belief in their abilities to be actively involved and coordinate schooling for their child.

This sentiment is encapsulated in a quote from a mother: “We acquired substantial knowledge … felt more confident, and understood our expectations … This knowledge was absolutely vital before initiating cooperation with the school” (Parent 11).

Parents articulated that the PIPP-RDs seemed to bolster their ability to influence the children’s schooling effectively. This suggest that this intervention program may have empowered parents and augmented their self-efficacy. Overall, most participants appear to have undergone a process of knowledge acquisition and enhanced health literacy related to their child`s school situation.

## Discussion

### Main findings

This mixed methods research indicates that parents of children with RDs have a substantial need for additional information to facilitate the transition from kindergarten to primary school. The PIPP-RDs was highly valued by the parents that participated in the program, and appears effective for this target population. Parents expressed strong approval concerning the content, organization, and applicability. Both quantitative and qualitative findings suggest that the program successfully empower parents to advocate their children’s needs. Nonetheless, further investigation is needed to assess the program`s impact of the children’s educational experiences.

Previous studies have found that similar intervention program for parents of children with other disabilities [19, 38], as well as for parents in general [39], exert a positive impact of parental self-efficacy in supporting their children through transition to school. These studies also reported associations between program participation and heightened parental involvement in school-related activities. These findings resonate with the experiences reported by parents in our study.

The qualitative data from the individual interview offered nuanced understanding of the reasons behind the high approval ratings for the PIPP-RDs by the parents. The parents emphasized the significance of obtaining relevant information, receiving practical advices, and having the opportunity to reflect on their child`s need alongside other parents navigating similar situations. Furthermore, they depicted an enhanced sense of psychological fortitude, competence, preparedness, relation, and improved communication skills in interacting with school staff after participating in the PIPP-RDs. Enhanced parental awareness of their role and knowledge about potential opportunities and obstacles appear to have positively influenced the facilitation of school transition.

### Increased competence, preparedness and reduced stress

Research highlights the need for intervention program aimed at empowering parents of children with disabilities, including RDs, to enhance their health literacy, skills, and confidence in managing their children’s needs [22, 40–43]. Parents of children with RDs often bear a significant burden in addressing challenges, navigating service system, sourcing information about their child`s condition, and evaluating healthcare and community resources [44, 45]. By providing information about specific contact points for parent-school communication may have encouraged early collaborative relationships with the school. This is consistent with numerous studies [46, 47] emphasizing the benefits of early preparations for school transitions to secure timely support and adjustments.

The parents reported feeling more prepared and less anxious about their child`s school transition after attending the PIPP-RDs, which is significance since increased parental worries have been associated with suboptimal child outcomes [39, 47]. Conversely, parents’ confidence and relaxation can contribute to smoother school adjustments for the children [39, 47]. Previous research has also identified feelings of resignation, anxiety, and perceived lack of support among parents of children with RDs [48, 49]. Our findings suggest that the PIPP-RDs program, by allowing parents to share experiences with others in similar situations, may help to alleviate such worries and feelings of isolation.

The group-discussions and informal social gathering may have provided parents with a shared sense of identity, mutual support, and a sense of community. Other studies have shown that group-based interventions for parents of preschool children can boost parental self-efficacy [27]. Encouragement from peers in similar situations may help parents overcome self-doubt and focus more effectively on their parental roles [50]. Some parents in our study also reported a reduction in stress levels after participating in the PIPP-RDs. This is in line with the study of Gómez-Zúñiga et al. [42], indicating that communication between the parents of children with RDs may be important for alleviating the burden and challenges in daily life.

### Enhancement of communication skills and self-efficacy

Effective communication between parents and school staff is essential for promoting optimal educational experience for children with RDs, as supported by empirical evidence [1, 12, 51]. Integrating pupils with RDs into regular schooling can present significant challenges, including perceived gaps in teachers’ qualifications regarding diversity and inclusion [51, 52].

Our findings suggest that the PIPP-RDs program, which equips parents with comprehensive knowledge of the school system, practical advices, and strategies to overcome potential barriers, may enhance parents` resilience and adaptability.

After participating in PIPP-RDs, parents experienced a more equal communication with the school staff, suggesting an empowerment that might have positively impacted their children’s schooling experience. The positive feedback from parents may reflect previously unmet needs for support, rather than solely the specific content of the PIPP-RDs program. While our study did not compare PIPP-RDs to other initiatives, it focused exclusively on the perceived impact of this particular program.

Nevertheless, the provision of pertinent information, the opportunity to interact with other parents navigating similar circumstances, the emphasis on recognizing their child`s strength and needs, and the provision of ample time for preparation and planning for the onset schooling were identified as beneficial components by the participants. Based on our findings, we propose that similar preschool programs, encompassing lectures, group discussion, information dissemination, and informal social interaction should be extended to other parents of children with RDs. Moreover, future studies should incorporate the perspectives of teachers regarding home-school collaboration to provide a more holistic understanding of this dynamic.

## Strengths and limitations

The current study`s strength rest primarily on the utilization of multi data sources, lending a comprehensive and robust portrayal of research questions. Additionally, the harmonious findings from both quantitative and qualitative aspects of the research bolstered the reliability of the conclusion drawn. A lack of discordance between the two data sources reinforces the credibility of our results. Furthermore, the survey`s design, allowing for anonymous participation, likely facilitated candid responses from the parents, thereby reducing the potential for response bias or the echo effect, where participants might tell researchers what they believe is desired. Our objective was to cultivate a more holistic and multidimensional understanding of a complex phenomenon such as the parents’ experience of the utility of participating in the PIPP-RDs. However, this intricate exploration necessitates further examination in larger studies with a greater number of participants.

Conversely, the study`s limitation include its lack of generalizability to parents of children with RDs due to several factors. These factors encompass a small sample size, local context, restriction to a limited number of RDs, selection bias originating from voluntary participation, potential echo effects in the interviews. Furthermore, the “Hawthorne effect” can introduce a form of bias as parents may improve or modify behaviour simply because they know they are part of this study. Regarding the survey component of the research, the lack of validated measurement tools tailored to the assessment of parental outcome led to the development of a study-specific questionnaire. This introduces the possibilities of misinterpretation of questions by the parents. However, the use of multiple data source mitigates this issue, thereby enhancing the study`s reliability.

Due to the small sample size, the study did not warrant advanced statistical analysis, therefore, we only performed descriptive analysis. Additionally, it is crucial to acknowledge the study`s contextual limitation. Being situated exclusively within Norway, the findings may not extend to different cultural contexts of school systems. Other limitations encompass the absence of school staff perspectives and the direct impact on children with RDs.

## Conclusion

This study suggests that parents of preschool children with RDs exhibit unmet needs for information and knowledge concerning the possibilities and potential obstacles involved in facilitating their children`s education. Participation in the PIPP-RDs appears to enhance parental preparedness and self-efficacy, thus enabling parents to better address the challenges associated with their child`s conditions. Parents also reported that the PIPP-RDs bolster their strategies for fostering effective school-home collaboration.

Programs akin to the intervention program could prove beneficial for other parents of children with RDs, as well as parents of children with other types other types of disabilities. Initiatives aimed at empowering the parents that participated in this study to adeptly navigate the school system are likely to yield similarly favourable results. Preschool intervention program, tailored for parents of children with RDs should be further developed and investigated across different cultural contexts and geographical regions. Considerable effort should be devoted to fostering a more robust evidence base for the successful implantation of such parent intervention programs.

The potential for international collaborative studies utilizing similar intervention program is vast. Such collaborations could enhance our understanding of parental needs and the potential impacts of these programs. Cultural adaptations may be necessary, but it is reasonable to expect that most parents of children with RDs could derive significant benefits from participating in such intervention programs.

### Electronic supplementary material

Below is the link to the electronic supplementary material.

Supplementary Material 1

Supplementary Material 2

## Data Availability

The dataset (transcribed interviews) from the qualitative individual interviews and the dataset from the web-study are available, but restrictions apply to the availability of these data, which are used under licence for the current study and are not publicly available. The data concerns a small groups of parents of children with rare diseases who participated in the PIPP-RDs. Both the qualitative interviews and survey data are confidential and sensitive with personal data involved. The data, are however available on reasonable request to the corresponding author (Gry.velvin@sunnaas.no).

## References

[1] Darretxe L, Gaintza Z, Monzon-Gonzalez J. A systematic review of research into rare diseases in the educational sphere. Academic Journals, Educational Research and review. 2017;12(10):589–594. 10.5897/ERR2017.3186.

[2] Slade A, Isa F, Kyte D, Pankhurst T, Kerecuk L, Ferguson J (2018). Patient reported outcome measures in rare diseases: a narrative review. Orphanet J Rare Dis.

[3] Global Genes. Rare diseases: facts and statistics Accessed 2 Nov 2017- Allies in Rare Disease - Global Genes Allies in Rare Disease - Global Genes.

[4] Wakap SN, Lambert DM, Olry A, Rodwell C, Gueydan C, Lanneau V (2020). Estimating cumulative point prevalence of rare diseases: analysis of the Orphanet database. Eur J Hum Genet.

[5] United Nation Resolution. General Assembly: Resolution adopted by the general Assembly 16 December 2021. Addressing the challenges of persons living with a rare disease and their families. On report of the third Committee. 76/132. 5 January 2022. N2135824.pdf (un.org), Final-UN-Text-UN-Resolution-on-Persons-Living-with-a-Rare-Disease-and-their-Families.pdf(rarediseasesinternational.org).

[6] Dellve L, Samuelsson L, Tallborn A, Fasth A, Hallberg LR (2006). Stress and well-being among parents of children with rare diseases: a prospective intervention study. J Adv Nurs.

[7] von der Lippe C, Neteland I, Feragen KB (2022). Children with a rare congenital genetic disorder: a systematic review of parent experiences. Orphanet J Rare Dis.

[8] Belzer LT, Wright SM, Goodwin EJ, Singh MN, Carter BS (2022). Psychosocial considerations for the child with Rare Disease: a review with recommendations and calls to action. Child (Basel).

[9] Valcárcel-Nazco C, Ramallo-Fariña Y, Linertová R, Ramos-Goñi JM, García-Pérez L, Serrano-Aguilar P. Health-Related Quality of Life and Perceived Burden of Informal Caregivers of patients with Rare Diseases in selected european countries. Int J Environ Res Public Health. 2022:5;19(13):8208. 10.3390/ijerph19138208. 10.3390/ijerph19138208 PMC9266302 35805867

[10] Knight AW, Senior TP (2006). The common problem of rare disease in general practice. Med J Aust.

[11] Uhlenbusch N. Perceived burden in dealing with different rare diseases: a qualitative focus group study. BMJ Open. 2019;9(12). 10.1136/bmjopen-2019-033353. 10.1136/bmjopen-2019-033353 PMC6937088 31888936

[12] Foster M, Adama E, Arabiat D, Runions K, Vithiatharan R, Zgambo M, Lin A (2022). Parents’ experiences of children with a rare disease attending a mainstream school: Australia. J Pediatr Nurs.

[13] Paz-Lourido B, Negre F, Iglesia B, VergerS (2020). Influence of schooling on the health-related quality of life in children with rare diseases. Health and Quality of Life Outcome.

[14] Svendby EB, Dowling F (2013). Negotiating the discursive spaces of inclusive education: narratives of experience from contemporary physical education. Scandinavian J Disabil Res.

[15] Han KG (2008). A study on the characteristics and educational support for children with rare diseases. Korean J Phys Multiple Health Disabil.

[16] Lee HH, Lee KM (2014). Students diagnosed with congenital vascular malformation as a form of rare diseases and their parents` experiences and needs. J Special Educ.

[17] Lin J, Lin L, Hung W (2013). Reported numbers of patients with rare diseases based on ten-year longitudinal national disability registries in Taiwan. J if Res Dev Disabil.

[18] Watson G, Sanders-Lawson ER, McNeal L (2012). Understanding parental involvement in American Public Education. Int J Humanit Social Sci.

[19] Leung C, Lam T, Yau S, Tsang S (2016). The effect of parent education program for preschool children with developmental disabilities: a randomized controlled trial. J Res Dev Disabil.

[20] Đurišić M, Bunijevac M, editors. (2017). Parental Involvement as an Important Factor for Successful Education. ceps Journal. 2017;7(3). DOI:EJ1156936.pdf (ed.gov).

[21] OECD 2012/13. Better policies for better life. Education GPS. The world of education at your fingertips. Updated February 2021. Review education policies - Education GPS - OECD: Parental involvement.

[22] Willis CE, Reid S, Elliott C, Nyquist A, Jahnsen R, Rosenberg M, Girdler S (2019). It`s important that we learn too. Empowering parents to facilitate participation in physical activity for children and youth with disabilities. Scand J Occup Ther.

[23] Education Act Norway. Act relating to primary and secondary education and training. the Education Act) - Lovdata; 1998.

[24] TRS National Resource Centre for Rare Disorders. : TRS National Resource Centre for Rare Disorders - Sunnaas sykehus.

[25] Amendum SJ, Liefreund MD (2018). Situated learning, professional development, and early reading intervention. A mixed methods study. J Educational Res.

[26] Bauer-Ramanzani C, Graney JM, Marshall HW, Sabieh C (2016). Flipped Learing in TESOL, Definition, Approaches and implementation. TESOL J.

[27] Wittkowski A, Dowling H, Smith DM (2016). Does engaging in a Group-based intervention increase parental self-efficacy in parent of Preschool Children. A systematic review of the current literature. J Child Fam stud.

[28] Questback website. 2020 Questback platforms: https://insightplatforms.com/platforms/questback/.

[29] von Elm E, Altman DG, Egger M, Pocock SJ, Gotzsche PC, Vandenbroucke JP (2008). Strengthening the reporting of observational studies in epidemiology (STROBE) statement: guidelines for reporting observational studies. J Clin Epidemiol.

[30] Braun V, Clarke V (2006). Using thematic analysis in psychological. Qualitative Res Psychol.

[31] Tong A, Sainsbury P, Craig J (2007). Consolidated criteria for reporting qualitative research (COREQ): a 32-item checklist for interviews and focus groups. Intern J Qual Health Care.

[32] Epstein JL (1995). School/family/community partnerships: caring for the children we share. Phi Delta Kappan.

[33] Powell DS, Batsche CJ, Ferro J, Fox L, Dunlap GA, Strength-Based (1997). Approach in support of Multi-Risk families: principles and issues. Top Early Child Special Educ.

[34] Nutbeam D, Levin-Zamir D, Rowlands G (2018). Health literacy in Context. Int J Environ Res Public Health.

[35] Nutbeam D (2000). Health literacy as a public health goal: a challenge for contemporary health education and communication strategies into the 21st century. Health Promot Int.

[36] Røykenes K (2008). Method triangulation – a methodological minefield or an enrichment of phenomena? (metodetriangulering – et metodisk minefelt eller en berikelse av fenomener). Sykepleien Forskning.

[37] Gutterman TC, Fetters MD, Creswell JW (2015). Integrating quantitative and qualitative results in health science mixed methods research through joint display. Ann Fam Med.

[38] Lau EX, Rapee RM, Coplan RJ (2017). Combining child social skills training with a parent early intervention program for inhibited preschool children. J Anxiety Disorder.

[39] Giallo R, Treyvaud K, Matthews J, Kienhuis M (2010). Making the transition to Primary School: an evaluation of a transition program for parents. Australian J Educational Dev Psychol.

[40] Adair B, Ullenhag A, Keen D, Granlund M, Imms C (2015). The effect of interventions aimed at improving participation outcomes for children with disabilities: a systematic review. Dev Med Child Neurol.

[41] Stenberg U, Haaland-Øverby M, Koricho AT, Trollvik A, Kristoffersen LGR, Dybvig S, Vågan A (2019). How can we support children, adolescents and young adults in managing chronic health challenges? A scoping review on the effects of patient education interventions. J Health Expect.

[42] Gómez-Zúñiga B, Moyano RP, Fernández MP, Oliva AG, Ruiz MA (2019). The experience of parents of children with rare diseases when communicating with healthcare professionals: towards an integrative theory of trust. Orphanet J Rare Dis.

[43] Pelentsov LJ, Laws TA, Esterman AJ (2015). The supportive care needs of parents caring for a child with a rare disease, scoping review. Disabil Health J.

[44] Baumbusch J, Mayer S, Sloan-Yip I (2018). Alone in a crowd? Parents of children with rare Diseases ‘Experiences of navigating the Healthcare System. Genet Couns.

[45] Schieppati A, Henter JI, Daina E, Aperia A (2008). Why rare diseases are an important medical and social issue. Lancet.

[46] Driessen G, Smith F, Sleggers P (2005). Parental involvement and educational achievement. Br Educational Res.

[47] Piskur B, Beurskens AJ, Jongmans MJ, Ketelaar M, Norton M, Frings CA, Hemmingsson H, Smeets RJ. (2012). Parents’ actions, challenges, and needs while enabling participation of children with a physical disability: a scoping review. BMC Pediatric. 2012:12:177. 10.1186/1471-2431-12-177. 10.1186/1471-2431-12-177 PMC3538071 23137074

[48] Anderson M, Elliott EJ, Zurynski AZ (2013). Australian families living with rare disease: experiences of diagnosis, health services use and needs for psychosocial support. Orphanet J Rare Dis.

[49] Dellve l, Samuelsson L, Tallborn A, Fasth A, Hallberg LRM. (2006). Stress and wellbeing among parents of children with rare diseases: a prospective intervention study. Journal of Advanced Nursing. 2006;53(4):392–402. 10.1111/j.1365-2648.2006.03736.x. 10.1111/j.1365-2648.2006.03736.x 16448482

[50] Banduras A. Cognitive function in Self-Efficacy: The Exercise of Control. Freeman: New York. 1997:; Chap. 6, 212–258.

[51] Alston-Abel NL, Berninger V (2018). Relationships between Home literacy Practices and School Achievement: implications for Consultation and Home-School collaboration. J Educ Psychol Consult.

[52] Linertová R, González-Guadarrama J, Serrano-Aguilar P, Posada-De-la-Paz M, Péntek M, Iskrov G, Ballester M (2019). Schooling of children with Rare Diseases and disability in Europe. Int J Disabil Dev Educ.

