# Supplementary material for: Parental Intervention Program for Preschool children with Rare Diseases – a mixed methods evaluation of parents’ experiences and utility
Source: Orphanet J Rare Dis. 2023 Oct 17;18:327. doi: 10.1186/s13023-023-02935-8 (PMC10583464; doi:10.1186/s13023-023-02935-8)
Supplement: Supplementary file 1 — Supplementary Material 1 [file 13023_2023_2935_MOESM1_ESM.docx]

**The Questionnaire for evaluating parent` perception and utility of participate in the PIPP-RDs**

| 1. **What is your age?** |  |
| --- | --- |
| 20-30 years |  |
| 31-40 years |  |
| 41-50 years |  |
| 51 years or older |  |
| 1. **What is your gender?** |  |
| Woman |  |
| Male |  |
| Others |  |
| 1. **Place of Residence?** |  |
| Large City (population greater than 50,000) |  |
| Small Town (population less than 50,000 |  |
| Larger Urban Area (population greater than 20,000) |  |
| Smaller Urban Area (population less than 20,000) |  |
| Rural area/village |  |
| 1. **How many schoolchildren do you have?** |  |
| 1 children |  |
| 2 children |  |
| 3 children |  |
| 4 children or more |  |
| 1. **How many adults from your family participated in this program?** |  |
| 1 parent |  |
| 2. parents |  |
| **How useful do you think the intervention was according to preparation for the start of school?** |  |
| 1. **Information and collaboration with the school staff and teachers*** |  |
| - - Very useful |  |
| - - Useful |  |
| - - Don`t know |  |
| - - Not useful |  |
| - - Useless |  |
| 1. ***Information and collaboration with other professionals**** |  |
| - - Very useful |  |
| - - Useful |  |
| - - Don`t know |  |
| - - Not useful |  |
| - - Useless |  |
| 1. ***To enhance more knowledge about my child`s needs**** |  |
| - - Very useful |  |
| - - Useful |  |
| - - Don`t know |  |
| - - Not useful |  |
| - - Useless |  |
| 1. ***Preparing transition and facilitate accommodations for my child`s needs at school?**** |  |
| - - Very useful |  |
| - - Useful |  |
| - - Don`t know |  |
| - - Not useful |  |
| - - Useless |  |
| 1. ***To improve collaboration between the school and kindergarten during this transition phase? **** |  |
| - - Very useful |  |
| - - Useful |  |
| - - Don`t know |  |
| - - Not useful |  |
| - - Useless |  |
| 1. ***To what extent was the intervention helpful in making you feel less stressed and more confident about how the school start would be for your child? **** |  |
| - - Very useful |  |
| - - Useful |  |
| - - Don`t know |  |
| - - Not useful |  |
| - - Useless |  |
| 1. **Did you as parents, contact the school before the start of school based on the information/knowledge you gained from participating in the intervention program? *** |  |
| - - Yes,   - Please explain?..... |  |
| - - No,   - Please explain |  |
| 1. **Do you believe that participation has contributed to the preparations for the start of school going more easier than if you had not participated? *** |  |
| - - Yes,   - Please explain why?..... |  |
| - - No,   - Please explain why not?..... |  |
| 1. **Is it likely that you, as parents, have better and(or more contact with the school (after the start of school) as a result of participating in the intervention- compared to if you had not participated?*** |  |
| - - Yes,   - Can you explain why…..? |  |
| - - No,   - Please explain |  |
| **How useful do you think the intervention was for the school start?** |  |
| 1. ***Collaboration with other health service**** |  |
| - - Very useful |  |
| - - Useful |  |
| - - Don`t know |  |
| - - Not useful |  |
| - - Useless |  |
| 1. ***Facilitating transition in the school situation*** |  |
| - - Very useful |  |
| - - Useful |  |
| - - Don`t know |  |
| - - Not useful |  |
| - - Useless |  |
| 1. ***Home-school collaboration*** |  |
|  |  |
|  |  |
|  |  |
|  |  |
|  |  |
| 1. ***Communications about your child`s needs**** |  |
| - - Yes,   - Explain.. |  |
| - - No,   - Explain… |  |
| 1. **Where there any of the topics from the intervention that you think were particularly useful in preparing the start of school and after your child had attended the school? See issues below.** |  |
| - 1. ***Expectations and possibilities to school start*** |  |
| - - Very useful |  |
| - - Useful |  |
| - - Don`t know |  |
| - - Not useful |  |
| - - Useless |  |
| - 1. ***Having a child with disability, group discussions*** |  |
| - - Very useful |  |
| - - Useful |  |
| - - Don`t know |  |
| - - Not useful |  |
| - - Useless |  |
| - 1. ***Physical Education – a subject for Everyone*** |  |
| - - Very useful |  |
| - - Useful |  |
| - - Don`t know |  |
| - - Not useful |  |
| - - Useless |  |
| - 1. ***How to inform others (about my child`s disease and needs)*** |  |
| - - Very useful |  |
| - - Useful |  |
| - - Don`t know |  |
| - - Not useful |  |
| - - Useless |  |
| - 1. ***Practical adaption and school start, group discussions*** |  |
| - - Very useful |  |
| - - Useful |  |
| - - Don`t know |  |
| - - Not useful |  |
| - - Useless |  |
| - 1. ***Starting school and Being Different*** |  |
| - - Very useful |  |
| - - Useful |  |
| - - Don`t know |  |
| - - Not useful |  |
| - - Useless |  |
| - 1. ***Navigating challenges of having a child with RDs, group discussions*** |  |
| - - Very useful |  |
| - - Useful |  |
| - - Don`t know |  |
| - - Not useful |  |
| - - Useless |  |
| - 1. ***Further plans for the child`s schooling*** |  |
| - - Very useful |  |
| - - Useful |  |
| - - Don`t know |  |
| - - Not useful |  |
| - - Useless |  |
| 1. **Did you experience benefit from meeting other parents in the same situation? *** |  |
| - - Yes,   - Explain……. |  |
| - - No,   - Explain….. |  |
| - 1. **Have you maintained contact with some of the other parents after the intervention was completed?** |  |
| - - Yes,   - Explain….. |  |
| - - No |  |
| 1. **Would you recommended other parents of preschool children with rare diseases to participate in a similar intervention program? *** |  |
| - - Yes,   - Please explain….. |  |
| - - No,   - Please explain….. |  |
| 1. **Do you feel that the intervention program met your need for information and do you believe it enhanced you knowledge about what how facilitate your child`s start of school? *** |  |
| - - Yes,   - Please explain…. |  |
| - - No,   - Please explain…… |  |
| - 1. **Did the content of the PIPP-RDs satisfied your needs for supporting the transition process? *** |  |
| - - Yes,   - Can you describe…… |  |
| - - No   - Can you describe- why not ?.... |  |
| Thank you so much for answering these questions. | |
