# Supplementary material for: Parental Intervention Program for Preschool children with Rare Diseases – a mixed methods evaluation of parents’ experiences and utility
Source: Orphanet J Rare Dis. 2023 Oct 17;18:327. doi: 10.1186/s13023-023-02935-8 (PMC10583464; doi:10.1186/s13023-023-02935-8)
Supplement: Supplementary file 2 — Supplementary Material 2 [file 13023_2023_2935_MOESM2_ESM.docx]

**Interview Guide for evaluation of the PIPP-RDs:**

**(It is a detailed interview-guide and should be used with flexibility)**

**Program: Introduction**

- The immediate thought about participating in PIPP-RDs,
- The possible significance of the children`s schooling.
- About the Program of PIPP-
  - Remember any issues of particular importance.
  - Issues missing in the program
  - Impact on collaboration with school
  - Impact on collaboration with other professionals or services
- Significance for information gathering, interpretation and use of information
  - Any thoughts about this
  - Practical examples
- Significance for communication with school staff and health services
  - Any thoughts about this?
  - Practical examples
- Significance for collaboration (particular school)
  - Any thoughts about this
  - Practical examples
- Significance of having a child with rare diseases
  - Any thoughts about this
  - Any practical examples
- Significance for preparing schooling
  - Any thoughts about this
  - Any practical examples
- Significance for the schooling
  - Any thoughts about this
  - The child`s experiences being at school.
  - Any practical examples
- Significance for the parents
  - Any thoughts about this
  - Any practical examples on how it might have influenced you.
- The content of PIPP-RDs.
  - What was of importance and what was of lesser relevance of PIPP-RDs? Was there anything the course lacked?
  - Do you have suggestions on how the PIPP-RDs could be improved?
- Suggestion to other parents in similar situations.
  - Any thoughts about this
  - Any practical advices

Thank you for answering these questions
